# Energetic limits: Defining the bounds and trade‐offs of successful energy management in a capital breeder

**DOI:** 10.1111/1365-2656.13312

**Published:** 2020-09-07

**Authors:** Courtney R. Shuert, Lewis G. Halsey, Patrick P. Pomeroy, Sean D. Twiss

**Affiliations:** ^1^ Department of Biosciences Durham University Durham UK; ^2^ Department of Life Sciences University of Roehampton London UK; ^3^ Scottish Oceans Institute University of St. Andrews St. Andrews UK

**Keywords:** capital breeding, energy management, grey seal, lactation, reproductive success

## Abstract

1. Judicious management of energy can be invaluable for animal survival and reproductive success. Capital breeding mammals typically transfer energy to their young at extremely high rates while undergoing prolonged fasting, making lactation a tremendously energy demanding period. Effective management of the competing demands of the mother's energy needs and those of her offspring is presumably fundamental to maximizing lifetime reproductive success.
2. How does the mother maximize her chances of successfully rearing her pup, by ensuring that both her pup and herself have sufficient energy during this ‘energetic fast’? While energy management models were first discussed in the 1990s, application of this analytical technique is still very much in its infancy. Recent work suggests that a broad range of species exhibits ‘energy compensation’; during periods when they expend more energy on activity, their bodies partially compensate by reducing background (basal) metabolic rate as an adaptation to limit overall energy expenditure. However, the value of energy management models in understanding animal ecology is presently unclear.
3. We investigate whether energy management models provide insights into the breeding strategy of phocid seals. Not only do we expect lactating seals to display energy compensation because of their breeding strategy of high energy transfer while fasting, but we anticipate that mothers exhibiting a lack of energy compensation are less likely to rear offspring successfully.
4. On the Isle of May in Scotland, we collected heart rate data as a proxy for energy expenditure in 52 known individual grey seal (*Halichoerus grypus*) mothers, repeatedly across 3 years of breeding. We provide evidence that grey seal mothers typically exhibit energy compensation during lactation by downregulating their background metabolic rate to limit daily energy expenditure during periods when other energy costs are relatively high. However, individuals that fail to energy compensate during the lactation period are more likely to end lactation earlier than expected.
5. Our study is the first to demonstrate the importance of energy compensation to an animal's reproductive expenditure. Moreover, our multi‐seasonal data indicate that environmental stressors may reduce the capacity of some individuals to follow the energy compensation strategy.

## Graphical abstract

Using grey seal mothers as a model species for extreme lactation constraints, this study demonstrates the importance of energy compensation to an animal's reproductive expenditure through the framework of energy management strategies. Futher, the multi‐seasonal data indicate that environmental stressors may reduce the capacity of some individuals to follow the energy compensation strategy

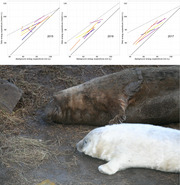

## 1 INTRODUCTION

Lactation is an energetically demanding period for mammals. Females must balance how they deploy their energy resources between maximising the relative amount of energy passed to their altricial offspring while ensuring they themselves stay alive and healthy. Maternal provisioning in phocid seals is an extreme example; most are capital breeders that sustain themselves and their single offspring exclusively on energy resources acquired prior to an acute lactation period undertaken while fasting for the duration of the pup rearing period (e.g. Schulz & Bowen, 2005). As an example, a grey seal (*Halichoerus grypus*) mother transfers over 18,000 kcal to its pup each day over an 18‐day lactation without acquiring any food (energy) herself (Iverson, Bowen, Boness, & Oftedal, 1993). Given the finite energy constraints associated with capital breeding, lactating females likely have a narrow margin of managing energy expenditure within which to achieve their objective of rearing their young while maintaining their own viability (Pomeroy, Fedak, Rothery, & Anderson, 1999). The demands of lactation in phocid seals typically result in a considerable increase in basal metabolic rate to well above that estimated from allometric equations (McLean & Speakman, 1999; Mellish, Iverson, & Bowen, 2000; Schweigert, 1993; Sparling, Speakman, & Fedak, 2006; Tedman & Green, 1987). Lactating seals are therefore expected to expend energy on themselves judiciously, for example by minimizing activity, as observed in many phocid species (Anderson & Fedak, 1987; Careau & Garland Jr., 2012; Collins et al., 2016; Hood & Ono, 1997; McLean & Speakman, 1999; Mellish et al., 2000; Thompson, Miller, Cooper, & Hammond, 1994). A seal may terminate lactation early if its body condition is poor or environmental conditions are particularly challenging to minimize the long‐term costs to itself so that longer term survival and future reproductive opportunities are favoured (Desprez, Gimenez, McMahon, Hindell, & Harcourt, 2018; McMahon, Harcourt, Burton, Daniel, & Hindell, 2016; Pomeroy et al., 1999). In addition, mothers that over‐expend in a breeding season risk missing the next breeding attempt, negatively impacting lifetime reproductive success (Pomeroy et al., 1999).

Considering animal energy expenditure in terms of different strategies of energy management is a helpful tool for understanding key energetic trade‐offs across a wide variety of ecological contexts (Careau & Garland Jr., 2012; Halsey et al., 2019; Portugal et al., 2016). Specifically, we can investigate how individuals balance background energy demands against the energy expenditure associated with daily activities and other auxiliary processes. Three models of energy management have been presented in the literature to quantify an animal's management of the competing demands of background energy expenditure, which includes aspects of body maintenance and milk production, with other energy costs such as activity (Careau & Garland Jr., 2012; Careau et al., 2013; Careau, Thomas, Humphries, & Réale, 2008; Halsey et al., 2019; Portugal et al., 2016; Ricklefs, Konarzewski, & Daan, 1996). The most intuitive and often implicitly assumed model of energy management is represented by the concept that an animal's daily background energy expenditures are simply added to other (auxiliary) energy costs resulting in its total daily energy expenditure. Thus, as background and auxiliary energy expenditures decrease or increase in response to demand, they do so independently of each other, and hence, this model is often referred to as ‘independent energy management’ (Halsey et al., 2019; Pontzer et al., 2014; Portugal et al., 2016). The ‘performance’ model of energy management (e.g. Careau, 2017; Halsey et al., 2019; Pontzer, 2015) is similar in that auxiliary and background expenditure is cumulative, but this model argues that high activity levels (auxiliary costs) require greater background energy expenditure to build or repair tissue in order to meet this higher daily energy expenditure demand. However, some evidence suggests that daily energy expenditure is constrained, even during energetically demanding periods, and as a result, individuals trade‐off auxiliary and background costs in order to maintain a cap on daily energy expenditures over time. This is known as ‘compensation energy management’ (Careau & Garland Jr., 2012; Careau et al., 2008; Halsey et al., 2019; Pontzer, 2015; Pontzer et al., 2016). Therefore, in terms of energy management, we might expect fasting phocid seal mothers to fit the energy compensation model, balancing energy spent on background processes, including milk production, against energy spent on activity and other auxiliary processes, in order to constrain daily energy expenditure (Mellish et al., 2000). Moreover, it is unclear what outcomes may be associated with grey seal mothers exhibiting a lack of energy compensation in their energy management strategy.

Individuals may be subject to different limits within which they can modulate background and auxiliary energy expenditure (due to constraints that vary across individuals such as local environment, body condition, size and age; Portugal, White, Green, & Butler, 2018; Signer, Ruf, & Arnold, 2011). Identifying the range of these bounds across a sample of a population would represent an envelope defining the limits within which a population of individuals trade‐off background and auxiliary energy expenditures without incurring negative energy balance as a consequence of excessive daily energy expenditure. Identifying envelopes of energy management for lactation across a population can therefore establish the typical limits within which individuals operate, and whether these vary across breeding seasons.

Studies of energy allocation have successfully generated measures of resting and activity‐specific energy expenditure by way of respirometry (Costa & Kooyman, 1982; Ward, Bishop, Woakes, & Butler, 2002; Wright, Metcalfe, Hetherington, & Wilson, 2014; Yeates, Williams, & Fink, 2007) and isotopically labelled injectables (Mellish, Iverson, Bowen, & Hammill, 1999; Rea et al., 2016; Reilly & Fedak, 1990; Rutishauser, Costa, Goebel, & Williams, 2010; Scrimgeour, Rollo, Mudambo, Handley, & Prosser, 1993), but these methods are typically restricted to providing finite snapshots of energy expenditure over time. Many techniques for measuring energy use are nearly impossible to implement continuously and would require multiple sampling events to obtain a useful temporal resolution of energy expenditure to investigate day‐to‐day trade‐offs. However, measurements of heart rate over extended periods in free‐ranging animals are a robust proxy of energy expenditure across a variety of activity levels (Green, 2011), and do not require multiple manipulations to determine daily fluctuations in energy expenditure and allocation. Minimum daily heart rate is a reliable proxy for background energy expenditure while mean daily heart rate is a proxy for daily energy expenditure (Halsey et al., 2019; Portugal et al., 2016). Thus, heart rate recordings enable investigation of the trade‐offs an animal makes between its background and auxiliary energy expenditure (Halsey et al., 2019; Portugal et al., 2016).

Here, we apply the heart rate method to investigate energy allocation in lactating grey seals through the lens of energy management strategies over multiple years, and how their energy management may relate to their immediate breeding success. We investigated energy management strategies of grey seal mothers over three successive breeding seasons by interpreting the relationship between daily energy expenditure (mean daily heart rate) and background energy expenditure (minimum mean daily heart rate) within and between individuals. Given the constraints already identified in this species, we expect that grey seals would be required to employ a compensation energy management strategy in order to maintain the extreme energy output of this brief lactation period. With data for multiple individuals per season, we were also able to quantify the limits of energy management within which the subpopulation operates, how these limits may vary between seasons and year‐on‐year differences in energy management between and within individuals. This culminated in an analysis comparing the energy management patterns of individuals that remained with their pups for an average lactation period against those that ended lactation much earlier than expected, indicating that achieving optimal energy management strategies is key to success in this species.

## 2 MATERIALS AND METHODS

### 2.1 Measuring heart rate

**FIGURE 1:**
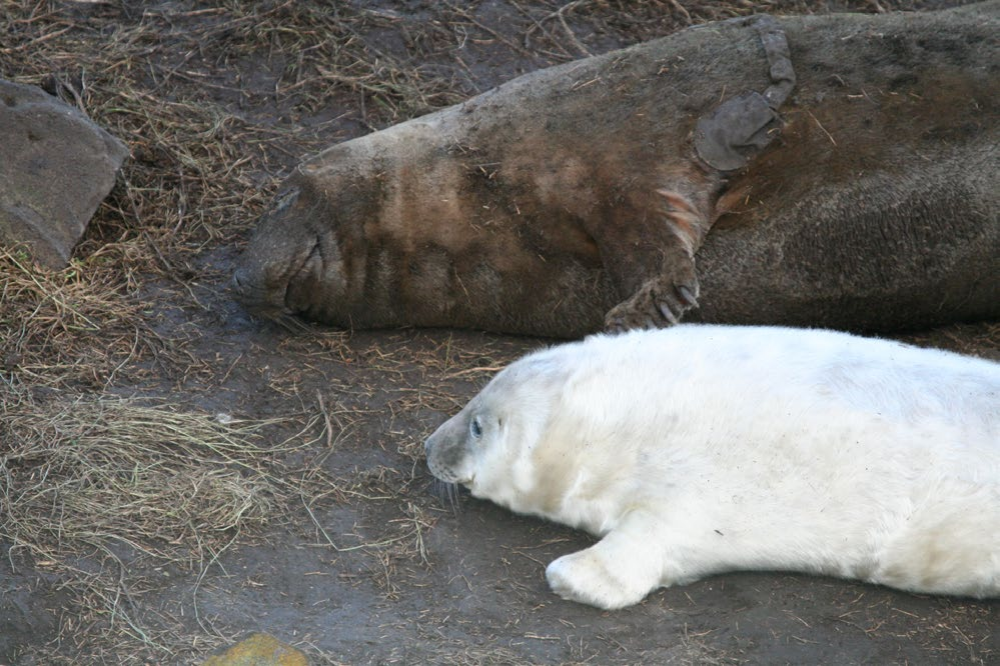
Heart rate monitor attached to a study female during the 2017 season, pictured here resting with her pup in view. Each heart rate monitor consisted of a central transmitter located dorsally with two electrode leads extending down each flank and attaching behind the fore‐flippers (demonstrated on one side of female pictured here; Photo: CR Shuert)

Fifty‐two females (*n* = 26 recaptured at least once for either two or three breeding seasons) and were equipped with heart rate monitors (FirstBeat Technologies, Ltd.; Twiss, Shuert, Brannan, Bishop, & Pomeroy, 2020) for the core duration of lactation in three breeding seasons between 2015 and 2017 (*n* = 87 lactation periods over all seasons; Figure 1). As continuous heart rate recordings were not possible, females were sampled for heart rate (*f_H_*, beats/min) when in range (50–200 m) of a portable recording station periodically moved around the colony during daylight hours (Twiss et al., 2020). This resulted in 1–6 hr of recordings per day for most individuals yielding daily samples of heart rate for every instrumented individual representing both resting and active periods. Segments of heart rate data (15‐min long) with <50% measurement artefacts were selected (see Appendix S1 in Supporting Information). In addition, data‐logging tri‐axial accelerometers were placed on the torso of 29 of these seals for concurrent periods over two of these breeding seasons (2016 and 2017; Technosmart Europe; Figure 1; Shuert, Pomeroy, & Twiss, 2018). Dynamic acceleration was derived from the raw measurements by calculating a running mean over a 3‐s window and subtracting the resultant values from the raw measurements in each of the three axes of data (Shepard et al., 2008). Vectorial dynamic body acceleration (VeDBA) was then calculated by taking the square root of the sum of squares of each axis of dynamic acceleration, and then smoothing (sVeDBA) over a 3‐s window to estimate activity levels over time (Fehlmann et al., 2017; Shuert et al., 2018; Stothart, Elliott, Wood, Hatch, & Speakman, 2016; Udyawer, Simpfendorfer, Heupel, & Clark, 2017; Wilson et al., 2019). Each 15‐min period of heart rate data was categorized as one of three levels of activity based on the resultant mean sVeDBA value: resting (largely inactive), low activity (little movement, alert) and high activity (continuous movement, such as locomotion/aggression; Shuert et al., 2018). See Appendix S1 for more information on the tagging procedures, accelerometers, heart rate monitors and artefact filtering procedures.

### 2.2 Energy management strategies

Basic heart rate statistics represent valuable proxies of energy expenditure relevant to energy management across a variety of taxa (Halsey et al., 2019; Portugal, Green, & Butler, 2007; Portugal et al., 2016, 2018). Specifically, minimum heart rate (min‐*f_H_*, calculated as the daily minimum value of mean heart rate from any 15‐min period) relates to energy expended on ‘background processes’, such as those relating to basal metabolism and other metabolic processes including milk production, tissue repair and changes in organ mass (‘background energy expenditure’; Careau, 2017; Halsey et al., 2019; Portugal et al., 2016, 2018). Mean daily heart rate (mean‐*f_H_*) reflects the energy expended over a day (‘daily energy expenditure’; Halsey et al., 2019; Portugal et al., 2016). The difference between these two measures (min‐*f_H_* subtracted from mean‐*f_H_*) is then attributed to all non‐background ‘auxiliary’ processes, perhaps principally activity (aux‐*f_H_*, ‘auxiliary energy expenditure’; Halsey et al., 2019; Portugal et al., 2016). The mean value for each heart rate proxy relating to background and daily energy expenditure (min‐*f_H_* and mean‐*f_H_*) was calculated for each day for each individual. While these heart rate proxies were not calibrated to metabolic rate directly in grey seals, min‐*f_H_* and mean‐*f_H_* have been positively correlated in every endothermic species measured so far as a robust proxy for metabolic rate (Green, 2011; Halsey et al., 2019). For vertebrate species with calibrated metabolic rate data, Portugal et al. (2016) demonstrated that these energy management strategy patterns hold and do not change when based on either heart rate‐ or metabolic rate‐based measurements.

The energy management strategy employed across individuals (i.e. as a population) and within individuals was modelled using the relationships between mean‐*f_H_* and min‐*f_H_*. Ordinary least squares (OLS) slopes were fit as detailed in Halsey et al. (2019) to describe energy management across individuals (mean value per individual) and within individuals (centralized values for each individual) across each year of study separately, using linear mixed‐effects models generated with the r package lme4 (Bates, Maechler, Bolker, & Walker, 2015). We also assessed whether individuals differed in their energy management strategy by comparing across‐ and within‐individual slopes for each year of study separately (Halsey et al., 2019; van de Pol & Wright, 2009); differences in within‐individual slopes across seasons were assessed through an analysis of variance (Quinn & Keough, 2002). Because we hypothesized that lactating grey seals will exhibit energy compensation, we anticipate that the relationship between mean‐*f_H_* and min‐*f_H_* will be <1. Across‐individual slopes were also computed with two different model II regressions to assess the degree of regression dilution influencing the slope values due to error in the *x*‐axis measurement (Halsey & Perna, 2019; Smith, 2009). The slopes fit by OLS were compared against slopes fit by ranged major axis (RMA) and standard major axis (SMA) using the r package lmodel2 (Legendre, 2018). In each case, statistical significance of the slopes was determined through permutation tests (Legendre & Legendre, 1998).

### 2.3 Limits of energy management

To estimate the upper limits of energy expenditure of the seals, a 95th quantile regression was performed across all data for the relationships of daily mean‐*f_H_* against daily min‐*f_H_*, for each breeding season of study independently (*τ* = 0.95; Cade & Noon, 2003; Cade, Terrell, & Schroeder, 1999; Horning, 2012), using the r package quantreg (Koenker, 2018). This quantile regression line and the line of unity between mean‐*f_H_* and min‐*f_H_* were used to define an envelope of energy management during lactation in this population of individuals, which was compared across each breeding season.

Throughout each breeding season, researchers surveyed the main breeding areas daily so that known seals were identified as soon as possible after coming ashore (Smout, King, & Pomeroy, 2020). When birth was not observed directly, it was estimated using age‐related mass and development characteristics (Kovacs & Lavigne, 1986). Mother/pup pairs were captured and weighed twice, near the start and end of lactation, to allow estimation of maternal postpartum mass directly after the pup is born, and maternal weaning mass at the end of lactation, with maternal absence defining weaning date (protocol in Pomeroy et al., 1999). Average normal lactation duration from 2004 to 2015 at Isle of May was 17.91 ± 0.35 days (*SE*; P. Pomeroy, unpubl. data). We aimed for a minimum of 10 days between captures (typically days 5 and 15 of 18) and 77 of the sampled breeding episodes conformed to this pattern (reached at least day 15 while still attending and nursing a pup: ‘normal’). However, some females terminated lactation sooner than expected and were observed departing the breeding colony on or before day 14 before equipment could be recovered (2016: *n* = 8; 2017: *n* = 2: ‘abnormal’). We compared these two groups in terms of individual energy management strategies. The probability of early cessation of lactation was modelled as a binary outcome using a generalized linear mixed effects model with a logit link as a function of centred within‐individual slopes and individual mean background energy expenditure (both modelled as a single predictor, additive or interaction), with individual seals included as a random effect (see Table S1 in the Supporting Information; Zuur, Ieno, Walker, Saveliev, & Smith, 2009). These logistic regression models were then compared based on Akaike information criterion, corrected for small sample size (AICc), as well as their relative weighting and overall deviance through AICc model selection methods (Burnham & Anderson, 2002). Initial model checks indicated that these data modelled were not overdispersed (Burnham, Anderson, & Huyvaert, 2011).

All animal procedures were performed under UK Home Office project license #60/4009 and conformed to the UK Animals (Scientific Procedures) Act, 1986. All research was approved ethically by the Durham University Animal Welfare Ethical Review Board as well as by the University of St. Andrews Animal Welfare and Ethics Committee.

## 3 RESULTS

### 3.1 Energy management strategies

**FIGURE 2:**
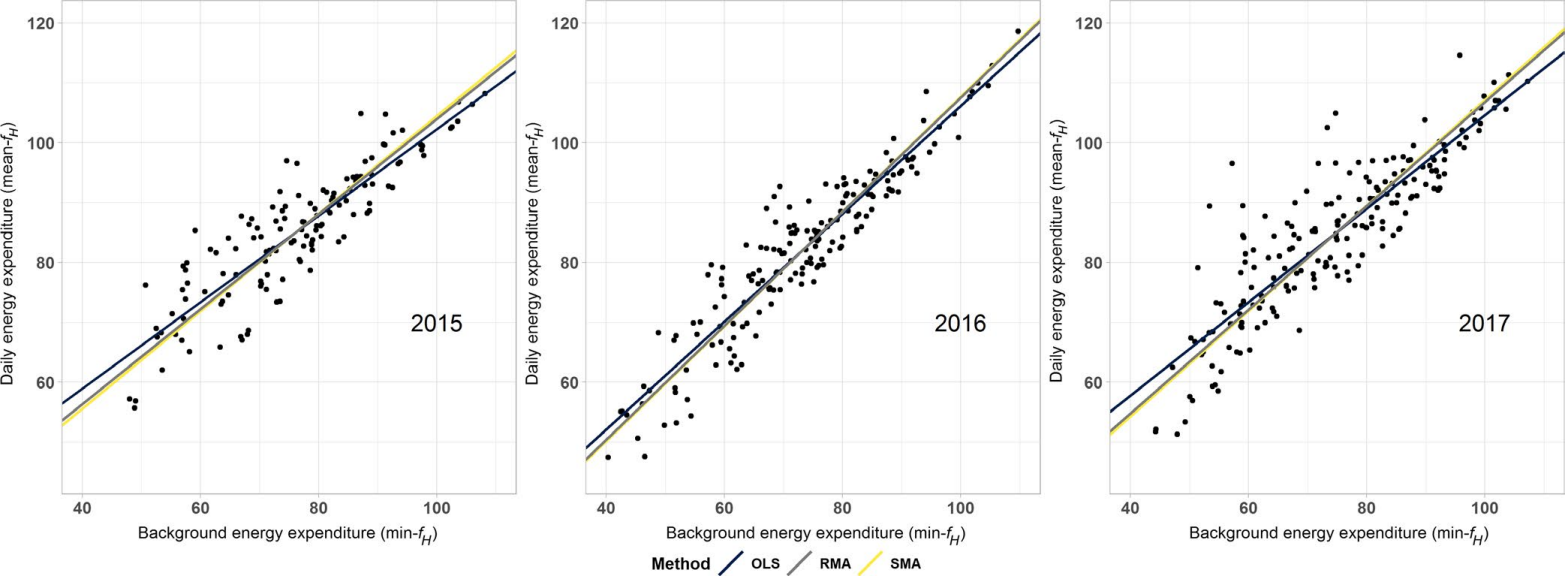
Grey seal energy management over lactation. Across‐individual relationships between daily values of mean‐*f_H_* (daily energy expenditure) against daily values of min‐*f_H_* (daily background energy). Relationships estimated using ordinary least squares (OLS; black line), ranged major axis (RMA; grey line) and standard major axis (SMA; yellow line) regression methods, illustrating varying degrees of regression dilution. OLS regression regularly appears to underestimate the true relationships between these variables since error likely exists in both sets of variables. As a population, a slope of <1 was found for each season of study indicating a compensation energy management strategy

Overall, across 51 female grey seals, 5,219 15‐min segments of heart rate data were analysed in three breeding seasons, representing various levels of activity (mean proportion of segments of heart rate data classified as ‘inactive’ = 0.433 ± 0.17, as ‘low activity’ = 0.363 ± 0.13 and as ‘high activity’ = 0.338 ± 0.16 across all individuals with accelerometers). One female in a single season was excluded from further analyses due to a lack of suitable heart rate data after artefact filtering. Both across and within individuals, energy compensation was exhibited by the seals, as indicated by mean‐*f_H_* ~ min‐*f_H_* slope values <1 for each year (across‐individual effect, OLS regression: 2015, 0.75 ± 0.05; 2016, 0.91 ± 0.04; 2017, 0.81 ± 0.05; within‐individual effect: 2015, 0.64 ± 0.04; 2016, 0.86 ± 0.05; 2017, 0.65 ± 0.05. In all cases, *p* < 0.001; Figure 2). Heart rate measures indicated that individuals with a greater daily energy expenditure than other individuals had relatively low background energy expenditures (across‐individuals finding) and individual seals partially compensated for increases in auxiliary energy expenditure with decreases in background energy expenditure and vice versa (within‐individuals finding). Model II regressions generated similar slope values to those fitted across individuals using the OLS approach (Table 1; Figure 2). In both 2015 and 2017, within‐individual slopes of mean‐*f_H_* ~ min‐*f_H_* were not different from across‐individual slopes on average (interaction of across‐ and within‐individual effects; 2015: −0.005 ± 0.005, *p* = 0.28; 2017: −0.008 ± 0.005, *p* = 0.12), but they did differ significantly in 2016 (−0.01 ± 0.005, *p* = 0.03). Within‐individual slopes were significantly different between years as determined through a two‐way analysis of variance (comparing slopes by year *F* = 54.1, *p* < 0.001). A Tukey pairwise comparison further highlights that within‐individual slopes were significantly steeper on average in 2016 than the other two seasons (Tukey HSD; 2016–2015: difference = 0.20, *p* < 0.001; 2017–2015: difference = −0.01, *p* = 0.68; 2017–2016: difference = −0.22 *p* < 0.001; see Table S2 in the Supporting Information).

**TABLE 1:** Population‐level patterns of energy compensation. Across‐individual slopes illustrating the relationship of mean‐*f_H_* against min‐*f_H_* fit to the centred value for each individual between breeding seasons for lactating female grey seals using three regression methods to account for dilution in both dimensions. Regression methods include ordinary least squares (OLS), ranged major axis (RMA) and standard major axis (SMA) and were fitted to the mean value for each female. In addition, slopes resulting from quantile regressions of these relationships are also included to demonstrate lactation envelopes of energy management across years. Confidence intervals (95%) are included in brackets next to each number (lower, upper)

Regression method	Slopes by season (across individuals)		
	2015	2016	2017
OLS	0.72^a^ (0.66, 0.79)	0.90^a^ (0.85, 0.95)	0.78^a^ (0.72, 0.84)
RMA	0.79^a^ (0.73, 0.87)	0.95^a^ (0.90, 1.01)	0.87^a^ (0.80, 0.93)
SMA	0.81 (0.73, 0.88)	0.96 (0.91, 1.01)	0.88 (0.83, 0.94)
Quantile *τ* = 0.95	0.54^b^ (0.49, 0.75)	0.77^b^ (0.69, 0.85)	0.53^b^ (0.47, 0.75)

^a^ Significance of slope (*p* < 0.01) based on 99 permutations of regression (SMA not able to be tested as such).

^b^ Significance of slope (*p* < 0.01) calculated based on confidence intervals by inverted rank test in Koenker and Machado (1999).

### 3.2 Limits of energy management

**FIGURE 3:**
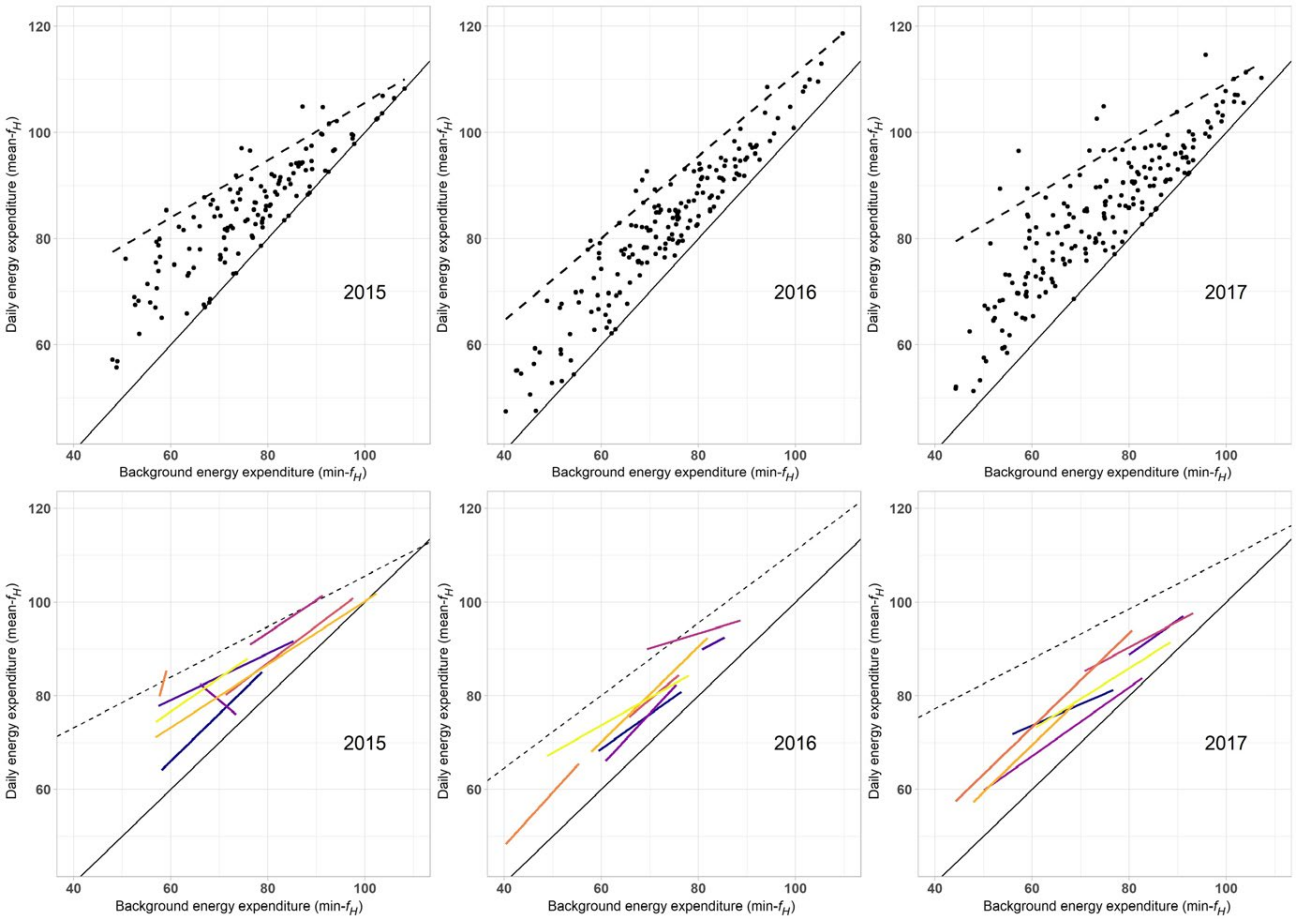
Envelopes of energy management. Broken black lines represent the 95th quantile regression (*τ* = 0.95) fitted for each year of study, representing the limits to energy compensation across the population between years, based on *f_H_* as an energy expenditure proxy. The solid black line represents the line of unity, indicating that daily energy expenditure (daily mean‐*f_H_*, beats/min) is solely contributed to by background energy expenditure (daily min‐*f_H_*, beats/min; as in Figure 2). Coloured lines represent the several females that were study subjects in all 3 years of study. These individuals (unique colours) occupy similar spaces within the envelope of energy management each breeding season as a result of relatively consistent background energy expenditure across seasons

**FIGURE 4:**
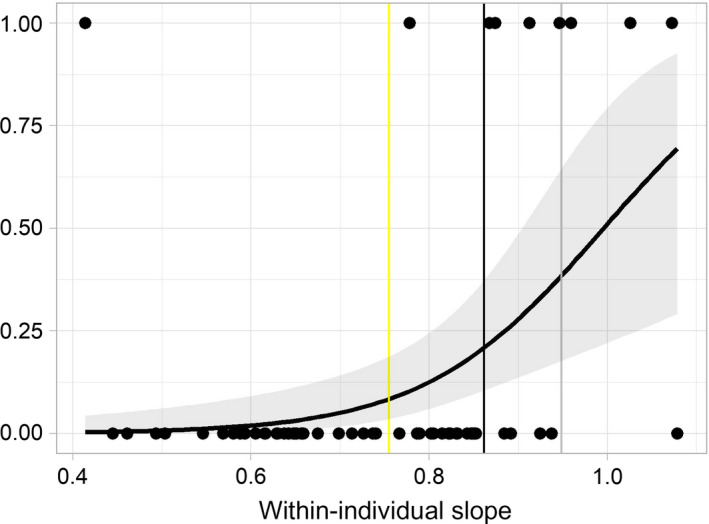
Within‐individual energy management strategy as a predictor of normal lactation behaviour. Individuals with slopes relating background (min‐*f_H_*) and daily energy expenditure (mean‐*f_H_*) approaching and surpassing 1 were statistically more likely to end lactation early compared to those with a slope of <1. The logistic regression is represented by the thick black line (with 95% confidence intervals in grey around), as a function of the individual data as black dots. Across‐individual slope (see Table 1) values are included for reference as vertical lines for each season (2015: yellow, 2016: grey, 2017: black)

Envelopes of energy management across individuals (bounded by the slope of unity and the 95th quantile line regression fit of mean‐*f_H_* against min‐*f_H_*) displayed a similar wedge‐like shape for each breeding season. As indicated by the shape of the wedge, the apparent scope for trading‐off background and auxiliary energy expenditures was largest for individuals that maintained lower background energy expenditure (min‐*f_H_*), thus allowing for a greater range of potential daily energy expenditures (mean‐*f_H_*). Despite the overall similarity of the shape of the envelope, 2016 was noticeably different exhibiting a steeper quantile slope than those for 2015 or 2017 (quantile regression, *τ* = 0.95; *p* < 0.001 for interaction for year and slope; Figure 3, Table 1). Although there was considerable variability in mean‐*f_H_* ~ min‐*f_H_* regression slopes between individuals within each year (Table 2; Figure 4; see also Table S2), there was within‐individual consistency across seasons in background energy expenditures (Table 2). Consequently, individual seals occupied similar regions within the energy management envelopes each season, suggesting they repeated strategies each season (Figure 3).

**TABLE 2:** Within‐individual patterns of energy compensation. Mean background energy expenditure (${\bar{x}}_{\text{min}}$; mean value of min‐*f_H_* for each season, beats/min) and slopes for the subset of female grey seals that were sampled over at least two seasons of study, illustrating the relationships between background and daily energy expenditure. Individuals highlighted in bold ceased lactation earlier than expected. Full results of within‐individual slopes can be found in Supporting Information Table S1

*ID*	2015		2016		2017	
	${\bar{x}}_{\text{min}}$	Slope	${\bar{x}}_{\text{min}}$	Slope	${\bar{x}}_{\text{min}}$	Slope
HG1	68.9	0.651	69.9	0.917	68.8	0.707
HG4	85.6	0.649			83.2	0.686
HG7	72.9	0.649	58.8	1.083		
HG9	66.0	0.648	74.4	0.920	79.2	0.662
HG12	77.2	0.648	83.0	0.850	85.1	0.685
HG16	102.6	0.649	**49.0**	**0.850**		
HG19	77.2	0.648			67.0	0.562
HG20	69.4	0.649	**67.3**	**1.012**	65.7	0.818
HG21			74.5	0.816	73.2	0.636
HG22	84.7	0.648	81.1	0.551	85.0	0.639
HG23			**68.1**	**1.093**	72.4	0.711
HG25	88.5	0.649			88.4	0.819
HG26	82.6	0.649			70.9	0.882
HG28	81.4	0.648			65.8	0.768
HG29			69.9	0.820	71.6	0.662
HG31	81.9	0.649	71.6	0.860	84.5	0.777
HG35			62.9	0.881	59.7	0.792
HG36	58.4	0.648	47.8	0.986	56.3	0.760
HG37	81.9	0.649	**61.7**	**1.161**		
HG39	72.7	0.649	69.3	0.912	57.8	0.837
HG40	65.5	0.648	65.4	0.701	72.5	0.711
HG41	62.7	0.648	**62.1**	**0.963**	75.6	0.350
HG43			**72.6**	**0.658**		
HG44			68.7	0.844	81.7	0.822
HG45			64.7	0.816	78.5	0.581
HG47			**67.2**	**0.667**	99.1	0.595

On average, individual females that ceased lactation earlier than expected for the colony (*n* = 9) had significantly steeper mean‐*f_H_* ~ min‐*f_H_* slopes (mean: 0.87 ± 0.24) than those that maintained lactation for the typical durations for the Isle of May of at least 15 days (*n* = 77; mean: 0.70 ± 0.12; Wilcoxon rank sum test, *W* = 198, *p* = 0.004; see Table 2). The females that ceased lactation earlier than expected often exhibited an alternate energy management strategy (slopes ≥1; see Table S2; Figure S1 in the Supporting Information). When modelled with a mixed‐effects logistic regression, within‐individual slope was found to be a significant predictor of ending lactation earlier than expected, carrying almost 70% of the overall model weight (within‐individual effect = 9.92 ± 3.07, *p* = 0.001; see Table S1). Mothers were more likely to cease lactation early as within‐individual slope approached, or exceeded, 1 (Figure 4). While our proxy for individual background energy expenditure varied across the population (Table 2), it was not a good predictor of early cessation of lactation. In all cases, min‐*f_H_* did not improve model deviance explained indicating that it was likely a pretending variable (Burnham & Anderson, 2002; see Table S1).

## 4 DISCUSSION

Grey seals, like most capital breeders, sustain themselves and their offspring exclusively on energy reserves acquired prior to the lactation period. Moreover, grey seals have some of the highest rates of mass transfer of extremely energy dense milk for a large mammal (Fedak & Anderson, 1982; Lydersen, Hammill, & Kovacs, 1995; Mellish, Iverson, & Bowen, 1999). Thus, to successfully rear their offspring, lactating grey seals must carefully balance the allocation of their energy reserves between the competing demands of their pup's needs and their own. Our data show that grey seal mothers balance the allocation of their energy reserves by compensating for periods when background energy demands are higher through decreasing their auxiliary energy costs, and vice versa (Figure 2). This strategy allows the proportion of the energy they are able to expend on their pup to be maximized while preventing over expenditure. In turn, the remaining daily energy budget available for other auxiliary processes such as activity is limited. Most behavioural studies of grey seals show that resting (inactivity) makes up the vast majority of a lactating female's activity budget, comprising at least 60% of daily time budgets during the breeding season (Anderson & Harwood, 1985; Fraser, Culloch, & Twiss, 2019; Kovacs, 1987; Shuert et al., 2018; Twiss, Caudron, Pomeroy, Thomas, & Mills, 2000). Our study confirms this; while we initially aimed to analyse heart rate data for a range of activity levels, the majority of heart rate traces represented periods of very low activity and inactivity.

Recent analyses by Halsey et al. (2019) suggest that for a diverse range of species, individual animals trade‐off their background or auxiliary metabolic rates in order to achieve energy compensation. Our data show that grey seals exhibit this within‐individual energy compensation too, agreeing with previous work by Mellish et al. (2000), but, unusually, they also exhibit energy compensation *across* individuals—individual seals that have relatively high background energy costs tend to also have relatively low auxiliary energy costs, and vice versa. In addition, our study shows that, typically, each seal exhibits its particular energy expenditure strategy consistently across breeding seasons. This means that, despite variation between individuals, a given individual tends to occupy similar energy management ‘space’, that is, similar magnitudes of daily and background energy expenditure, year on year (Figure 3). This combination of both within‐ and across‐individual energy compensation highlights the importance of judicious energy management within populations of grey seals during breeding, which we expect may well be the case for other capital breeding systems or species with inherently constrained life histories. Differing degrees of compensation are likely across capital breeding pinnipeds given the wide variety of lactation lengths and energy outputs represented (McDonald, Goebel, Crocker, Tremblay, & Costa, 2009; Schulz & Bowen, 2004, 2005; Sparling et al., 2006). While our study included experienced females, other studies have suggested that primiparous females often have shorter lactation bouts and may be more likely to be found in suboptimal locations on the periphery of the colony (Bowen, den Heyer, McMillan, & Iverson, 2015; Bowen, Iverson, McMillan, & Boness, 2006; Twiss et al., 2000). It is unclear how energy management strategies change with age and experience, but primiparous females may experience shorter lactation bouts because they are unable to exhibit energy compensation as their peripheral environment requires them to expend relatively large amounts of energy on activity than individuals in prime breeding locations (Twiss et al., 2000), but warrants further investigation.

Within our study, some individual females exhibited energy expenditure without compensation, most noticeably in 2016, where six females exhibited regression slopes of background and daily energy expenditure equal to or >1 (Figure 4). These atypical slopes are indicative of an independent or even performance energy management strategy. These mothers were statistically more likely to cease lactation early and depart the colony (compared to the typical 18 days of lactation seen at the Isle of May colony), consequently weaning their offspring much earlier than mothers who maintained energy compensation. This pattern was seen regardless of individual differences in background energy expenditures (Figure 4). Previous work has demonstrated that female seals may adjust their reproductive output between years, when faced with changing environmental conditions prior to the lactation period, in order to maximize lifetime reproductive output (Bowen et al., 2015; Desprez et al., 2018; Kalberer, Meise, Trillmich, & Krüger, 2018; McMahon et al., 2016; Pomeroy et al., 1999; Smout et al., 2020), but research has yet to reveal the mechanism for terminating lactation in grey seals (e.g. by investigating metabolite trends leading up to weaning; Mellish & Iverson, 2001; Watson, Pomeroy, Al‐Tannak, & Kennedy, 2020). Extended time spent applying a non‐compensatory energy strategy may result in a heightened energy debt, forcing those individuals to terminate their breeding attempt and possibly also skip the following breeding season to extend their recovery time (Pomeroy et al., 1999).

The envelopes of energy expenditure estimated in our study indicate the range of daily energy expenditure exhibited by lactating grey seals in the sampled population (Figure 3). It is possible that the upper boundary of the envelopes for energy management represents long‐term sustainable maximum metabolic rate during lactation. This idea is somewhat analogous to the concept of an animal's metabolic scope, where the upper limits of short‐term energy expenditure, sometimes referred to as maximum metabolic rate, are expressed as a multiple of basal metabolic rate (Binder et al., 2016; Bishop, 1999; Thurber et al., 2019). The width of these envelopes at any given value of background energy expenditure approximates the energy available for auxiliary energy expenditures for this population of individuals. Because the lower boundary of the envelope, which represents background energy expenditure being the entirety of daily energy expenditure, has a steeper slope than the upper boundary, individuals with lower background energy expenditures have a wider scope in which to vary their auxiliary energy expenditure (Figure 3). However, our results indicate that individuals with higher background energy expenditures were not necessarily more likely to cease lactation early, despite a narrower metabolic scope (see Table S1). This indicates that an individual's energy management strategy is more important than observed individual differences in background energy expenditure for determining the duration of lactation within the tight constraints of this capital breeding system.

The upper boundary of the envelope of energy management was markedly different in 2016 compared to the other two breeding seasons, having a much steeper slope (Figure 3). The 2016 breeding season, during which the majority of failed breeders within our study were recorded (Figure 4), was characterized by higher than average daytime temperatures and little rainfall (2015:9.37 ± 3.0°C, 133.6 mm total rain; 2016:11.15 ± 3.36°C, 35.8 mm total rain; 2017:10.94 ± 3.2°C, 40.5 mm total rain). These abiotic factors have previously been demonstrated as stressors for grey seals during lactation (Redman, Pomeroy, & Twiss, 2001; Stewart, Pomeroy, Duck, & Twiss, 2014; Twiss et al., 2002). Other studies indicate that the upper boundaries of metabolic scope decrease when temperatures are high (Farrell et al., 2008; Nilsson, Crawley, Lunde, & Munday, 2009; Pörtner, 2002; Sandblom, Gräns, Axelsson, & Seth, 2014). The apparent shifts of this envelope for energy management observed alongside these extrinsic pressures certainly warrant further investigation. External factors such as changing weather patterns affecting the scope for energy management may provide a mechanism linking environmental change to population change via variable reproductive outcomes.

For a long time, researchers have surmised that judicious energy expenditure should be important to many animals, particularly in situations where energy availability is limited and output is high (McNab, 2002). Recently, energy compensation has been explored as a possible mechanism to limit daily energy costs. We show that not only are lactating grey seals typically employing energy compensation, but those individuals which fail to do so are more likely to prematurely abandon their pup. Energy compensation appears to be an important strategy for this capital breeder. Moreover, our multi‐seasonal data allude to the possibility that environmental stressors may reduce the capacity of at least some individuals to follow the energy compensation strategy, and future studies thoroughly investigating this premise may be instrumental in highlighting how changes to ambient conditions, whether natural or man‐made, can perturb an animal's energy management at crucial periods of its life history, in ways potentially deleterious to its reproductive success.

## AUTHORS' CONTRIBUTIONS

C.R.S., S.D.T. and P.P.P. conceived of the study, participated in the field work and collected data; C.R.S. performed data analyses with support from L.G.H., P.P.P. and S.D.T.; C.R.S. wrote the paper with input from all authors. All authors have read and approved of the final manuscript.

## Supporting information

Supplementary Material

## Data Availability

Data available from the Dryad Digital Repository https://doi.org/10.5061/dryad.8w9ghx3jn (Shuert, Halsey, Pomeroy, & Twiss, 2020).
