# Supplementary material for: Energetic limits: Defining the bounds and trade‐offs of successful energy management in a capital breeder
Source: J Anim Ecol. 2020 Sep 7;89(11):2461–72. doi: 10.1111/1365-2656.13312 (PMC7693042; doi:10.1111/1365-2656.13312)
Supplement: Supplementary file 1 — Supplementary Material [file JANE-89-2461-s001.doc]

**Title:** Energetic limits: Defining the bounds and trade-offs of successful energy management in a capital breeder

**Authors**: Courtney R. Shuert, Lewis G. Halsey, Patrick P. Pomeroy, Sean D. Twiss

**Supporting Information**

**Appendix S1:**

*Ethical statement*

All applicable international, national, and/or institutional guidelines for the care and use of animals were adhered to in this study. All animal procedures were performed under UK Home Office project license #60/4009 and conformed to the UK Animals (Scientific Procedures) Act, 1986. All research was approved ethically by the Durham University Animal Welfare Ethical Review Board as well as by the University of St. Andrews Animal Welfare and Ethics Committee.

*Tagging Procedures*

Adult female grey seals (*n* = 52) were handled with their pups on the Isle of May (56.1° N, 2.55° W) over 3 consecutive breeding seasons (October to December) between 2015 and 2017 initially at around day 5 of lactation. Each female was chemically immobilized with a mass-specific dose of tiletamine-zolazepam (‘Zoletil’, Virbac, U.K.; Pomeroy, Twiss, & Duck, 2000). Each female was equipped with a heart rate monitor (Twiss, Shuert, Brannan, Bishop & Pomeroy, 2020) and some females also received an accelerometer at this initial handling event. All telemetry devices were then removed prior to weaning at around day 15. Each heart rate monitor activity belt (FirstBeat Technologies Ltd.; see also Halsey et al., 2019; Twiss, Shuert, Brannan, Bishop & Pomeroy, 2020) was placed on the back with the central transmitter housing located behind the shoulders. Silver chloride electrodes were placed immediately posterior to the fore-flippers with ample leads extending down either flank. A small section of pelage was clipped to match the size of the electrode and held in place with a small plastic donut and cover plate packed with medical-grade electrogel. Accelerometers were placed on the midline, just forward of the heart rate transmitter, when applicable (*n* = 29) in 2016 and 2017 so that forward motion of the female aligned with a positive x-axis. Accelerometers were sampled at 25 Hz at ± 2 *g* in 2016 (*nind* = 7) and 50 Hz at ± 4 *g* in 2017 (*nind* = 18; AXY-Depth, Technosmart Europe, Italy), for a minimum of 6 days over lactation. In addition, 4 individuals were equipped with GPS-enabled accelerometers in the 2017 season (50 Hz ± 4 *g*; AXY-Trek, Technosmart Europe, Italy). Both the heart rate monitors and accelerometers were housed in custom-built ballistic nylon pouches and glued directly to the upper layer of dry pelage with super glue (Loctite, formula ‘422’; see also Shuert, Pomeroy, & Twiss, 2018). Each 15-min period of heart rate data was categorised as one of three levels of activity: resting (largely inactive but including slight movement; mean smoothed VeDBA < 0.0275), low activity (little movement while the animal is alert; mean smoothed VeDBA ranging 0.0275 – 0.05), and high activity (continuous movement, such as locomotion or aggression; mean smoothed VeDBA > 0.05; Shuert, Pomeroy, & Twiss, 2018).

*Sampling Heart Rate Data and Mother-pup Pairs* in situ

As part of a long-term demographic and behavioural study of grey seals in the UK, known grey seal mothers, including those in the present study, were closely monitored and surveyed daily. Most females were known from previous seasons and were identified when returning to the colony before parturition; females on this colony regularly return to similar pupping sites (Pomeroy, Twiss & Duck 2000; Twiss, Caudron, Pomeroy, Thomas, & Mills, 2000). Individuals were identified by flipper tags, brands and unique pelage patterns (Smout, King & Pomeroy 2011). Throughout each breeding season, researchers surveyed the main breeding areas daily so that known seals were identified as soon as possible after coming ashore (Smout, King, & Pomeroy, 2011). When birth was not observed directly, it was estimated using age-related mass and development characteristics (Kovacs & Lavigne 1986). Mother/pup pairs were captured and weighed twice, near the start and end of lactation to allow estimation of maternal postpartum mass directly after the pup is born, and maternal weaning mass at the end of lactation, with maternal absence defining weaning date (protocol in Pomeroy et al., 1999). Lactation duration from 2004-2015 at Isle of May was 17.91 ± 0.35d (mean ± SE; P. Pomeroy, unpublished data).  Mother-pup pairs were observed daily to monitor lactation progress and to gather behavioural data as part of a larger study (Shuert, Pomeroy, & Twiss, 2018, 2020; Twiss, Shuert, Brannan, Bishop & Pomeroy, 2020). Grey seal mothers wean pups abruptly by returning to sea. Weaning dates were considered as the first day that the pup was sighted without its mother.

While individuals were followed for focal video sampling (see Shuert, Pomeroy, & Twiss 2020), heart rate data were recorded in real-time (Twiss, Shuert, Brannan, Bishop, & Pomeroy, 2020). Focal seals were equipped with modified Firstbeat™ heart rate belts (Jyväskylä, Finland) that recorded and transmit inter-beat intervals (IBIs) with millisecond precision to a remote receiver (Twiss, Shuert, Brannan, Bishop, & Pomeroy, 2020). With this equipment, we were able to record heart rate in real time from all equipped individuals within 200 m of the receiver (Figure 1). While it was not possible to record all individuals simultaneously throughout the day, every effort was made to sample each female’s heart rate once a day. Logistics and weather occasionally disrupted collection, but nonetheless each female was effectively sampled periodically over the duration of lactation to capture any differences in metabolic output associated with the changing energy demands of lactation (Mellish, Iverson, & Bowen, 2000). Heart rate recordings were validated as part of a larger study by simultaneous measurements of electrocardiogram (ECG) recordings upon recapture of females (Brannan, 2017; Twiss, Shuert, Brannan, Bishop, & Pomeroy, 2020)

The IBI data provided by this system were corrected for extreme values using Firstbeat™ Sports software (v.4.5.0.2) which deletes spurious extra beats (extreme short IBIs) and interpolates for potentially missing beats (extreme long IBIs; Sami, Mikko, & Antti, 2004). IBI data were further filtered by highlighting invariable sequences of inter-beat intervals (two or more consecutive identical inter-beat interval values; flats) or monotonically changing inter-beat intervals (two or more identical non-zero changes in inter-beat interval values; stairs) using custom-designed R scripts (see Twiss*,* Shuert, Brannan, Bishop, & Pomeroy, 2020). Once heart rate traces were broken up into non-overlapping 15-minute segments, only those segments that had less than 50% of these flats and stairs were utilised to derive measures of minimum heart rate (min-*fH*, calculated as the daily minimum value of mean heart rate) and mean daily heart rate (mean-*fH*) for each individual in each breeding season. We specifically chose to analyse heart rate over 15-minute segments to mitigate any potential bias in calculating our metrics associated with energy management which would be influenced by periods of rest-associated apnoea on land during shorter time periods (Andrews et al., 1997; Boyd, Bevan, Woakes, & Butler, 1999; Boyd, Woakes, Butler, Davis, & Williams, 1995; Lester & Costa, 2006; Ponganis, Stockard, Levenson, Berg, & Baranov, 2006).

**Supplemental Results**

**Figure S1:** The contrasting energy management trajectories of seals that failed lactation compared to those that were successful. The best fit lines for the individuals that ceased lactation earlier than expected are highlighted in colour; other females are represented by grey lines. The solid and broken black lines denote the envelope for energy management, defined in Figure 3. Several individuals who ceased lactation earlier than expected were found show slopes greater than or equal to 1, indicating alternate energy management strategies.

**
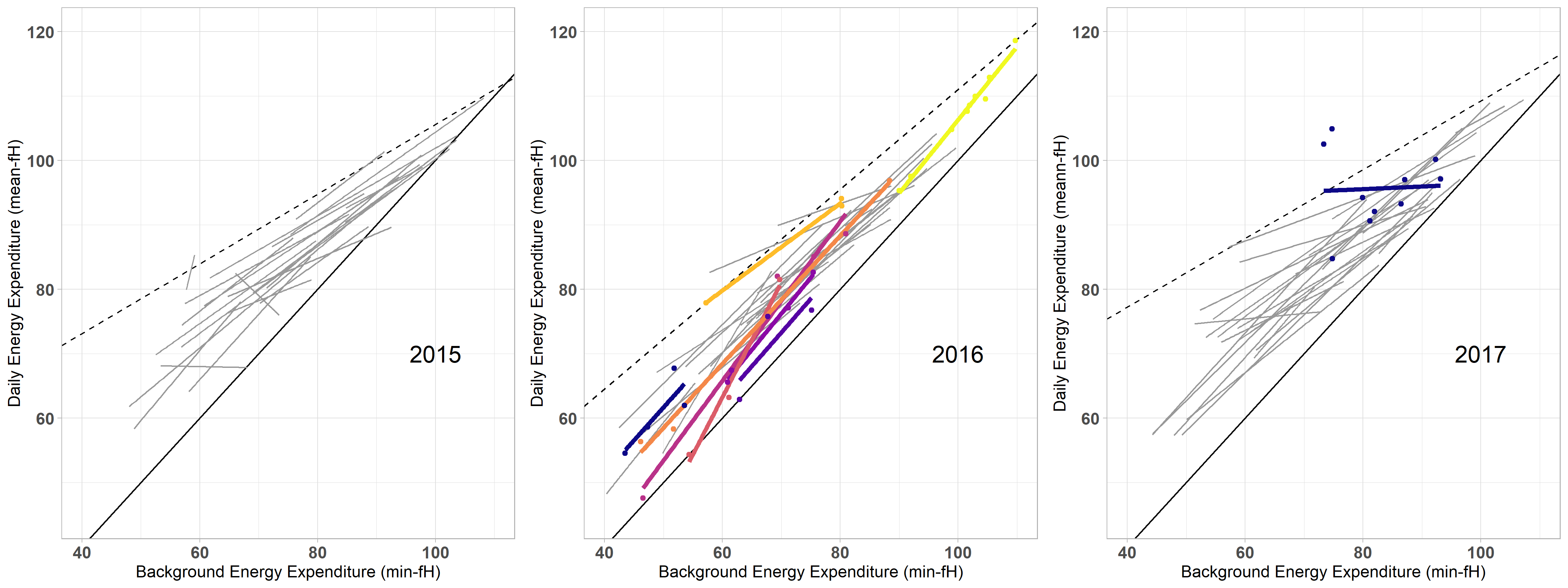
**

**Table S1:** Probability of ceasing lactation earlier than expected. Results for predicting the probability the female grey seals ‘fail’ (S/F – ceased lactation early and departed prior to day 15 of lactation), modelled as a generalized linear mixed effects model (with a logit link function) as a function of within-individual slope and individual mean background energy expenditure (*x̅ min-fH*). Each individual was included as a random effect. Models were assessed based on their relative Akaike Information Criterion, corrected for small sample size (Δ AICc), model weight, and deviance. Results from models with mean background energy expenditure were excluded from further analysis as they failed to improve model deviance to those not including this term.

| Model | *k* | Δ AICc | Weight | Deviance |
| --- | --- | --- | --- | --- |
| **S/F ~ Slope** | **3** | **0** | **0.688** | **44.194** |
| S/F ~ Slope + *x̅ min-fH* | 4 | 2.180626 | 0.231 | 44.176 |
| S/F ~ Slope* *x̅ min-fH* | 5 | 4.413982 | 0.075 | 44.156 |
| **S/F ~ Null** | **2** | **11.53093** | **0.002** | **57.871** |
| S/F ~ *x̅ min-fH* | 3 | 12.31387 | 0.001 | 56.508 |

**Table S2:** Within-individual slopes of mean-*fH* against min-*fH­* as well as aux-*fH­*against min-*fH* (though these represent complementary trends) and for lactating female grey seals determined from a linear mixed effects model for each year. Individuals highlighted in bold were found to end lactation earlier than expected (prior to 15 days post-partum).

|  | mean-*fH* against min-*fH­* | | | | | | aux-*fH­*against min-*fH* | | | | | |
| --- | --- | --- | --- | --- | --- | --- | --- | --- | --- | --- | --- | --- |
|  | 2015 | | 2016 | | 2017 | | 2015 | | 2016 | | 2017 | |
| *ID* | *x̅ min-fH* | *Slope* | *x̅ min-fH* | *Slope* | *x̅ min-fH* | *Slope* | *x̅ aux-f­H* | *Slope* | *x̅ aux-fH* | *Slope* | *x̅ aux-fH* | *Slope* |
| HG1 | 68.905 | 0.651 | 69.915 | 0.917 | 68.772 | 0.707 | 6.113 | -0.349 | 6.075 | -0.083 | 8.851 | -0.293 |
| HG2 |  |  | 75.692 | 0.818 |  |  |  |  | 7.583 | -0.181 |  |  |
| HG3 | 85.596 | 0.649 |  |  | 83.211 | 0.686 | 6.769 | -0.351 |  |  | 9.091 | -0.314 |
| HG4 |  |  | 82.493 | 0.796 |  |  |  |  | 7.196 | -0.203 |  |  |
| HG5 | 78.183 | 0.649 |  |  |  |  | 5.897 | -0.351 |  |  |  |  |
| HG6 |  |  | 76.452 | 0.745 |  |  |  |  | 8.299 | -0.255 |  |  |
| HG7 | 72.931 | 0.649 | 58.792 | 1.083 |  |  | 6.377 | -0.350 | 9.464 | 0.083 |  |  |
| HG8 | 66.609 | 0.651 |  |  |  |  | 9.360 | -0.349 |  |  |  |  |
| HG9 | 66.008 | 0.648 | 74.361 | 0.920 | 79.201 | 0.662 | 16.262 | -0.351 | 6.427 | -0.079 | 7.796 | -0.338 |
| HG10 | 91.238 | 0.649 |  |  |  |  | 8.374 | -0.351 |  |  |  |  |
| HG11 | 79.693 | 0.649 |  |  |  |  | 7.081 | -0.335 |  |  |  |  |
| HG12 | 77.168 | 0.648 | 82.955 | 0.850 | 85.106 | 0.685 | 10.428 | -0.352 | 8.133 | -0.150 | 7.416 | -0.315 |
| HG13 | 78.423 | 0.647 |  |  |  |  | 12.142 | -0.352 |  |  |  |  |
| HG14 | 91.138 | 0.649 |  |  |  |  | 4.574 | -0.351 |  |  |  |  |
| HG15 |  |  |  |  | 91.592 | 0.569 |  |  |  |  | 8.924 | -0.431 |
| HG16 | 102.601 | 0.649 | **49.037** | **0.850** |  |  | 0.000 | -0.351 | **11.672** | **-0.149** |  |  |
| HG17 | 57.769 | 0.650 |  |  |  |  | 10.333 | -0.349 |  |  |  |  |
| HG18 |  |  | **68.569** | **1.046** |  |  |  |  | **3.238** | **0.046** |  |  |
| HG19 | 77.193 | 0.648 |  |  | 66.967 | 0.562 | 10.121 | -0.351 |  |  | 14.226 | -0.438 |
| HG20 | 69.403 | 0.649 | **67.265** | **1.012** | 65.721 | 0.818 | 10.157 | -0.351 | **5.917** | **0.011** | 5.572 | -0.182 |
| HG21 |  |  | 74.493 | 0.816 | 73.189 | 0.636 |  |  | 8.048 | -0.184 | 11.670 | -0.364 |
| HG22 | 84.748 | 0.648 | 81.065 | 0.551 | 84.980 | 0.639 | 12.018 | -0.351 | 12.574 | -0.449 | 8.106 | -0.361 |
| HG23 |  |  | **68.071** | **1.093** | 72.367 | 0.711 |  |  | **7.747** | **0.092** | 7.863 | -0.289 |
| HG24 |  |  |  |  | **82.502** | **0.387** |  |  |  |  | **13.161** | **-0.613** |
| HG25 | 88.503 | 0.649 |  |  | 88.406 | 0.819 | 4.225 | -0.350 |  |  | 3.088 | -0.181 |
| HG26 | 82.631 | 0.649 |  |  | 70.947 | 0.882 | 8.901 | -0.351 |  |  | 5.905 | -0.118 |
| HG27 |  |  |  |  | 69.562 | 0.467 |  |  |  |  | 14.846 | -0.533 |
| HG28 | 81.390 | 0.648 |  |  | 65.775 | 0.768 | 10.031 | -0.351 |  |  | 8.144 | -0.232 |
| HG29 |  |  | 69.946 | 0.820 | 71.550 | 0.662 |  |  | 11.819 | -0.179 | 9.251 | -0.338 |
| HG30 | 65.713 | 0.649 |  |  |  |  | 12.703 | -0.351 |  |  |  |  |
| HG31 | 61.081 | 0.649 |  |  |  |  | 6.890 | -0.351 |  |  |  |  |
| HG32 | 81.914 | 0.649 | 71.637 | 0.860 | 84.508 | 0.777 | 6.611 | -0.350 | 8.978 | -0.140 | 0.000 | -0.223 |
| HG33 |  |  |  |  | 71.339 | 0.385 |  |  |  |  | 15.796 | -0.615 |
| HG34 |  |  |  |  | 62.722 | 0.558 |  |  |  |  | 12.906 | -0.442 |
| HG35 |  |  | 62.881 | 0.881 | 59.742 | 0.792 |  |  | 7.220 | -0.119 | 11.772 | -0.208 |
| HG36 | 58.409 | 0.648 | 47.838 | 0.986 | 56.254 | 0.760 | 24.209 | -0.352 | 9.045 | -0.013 | 13.237 | -0.240 |
| HG37 | 81.907 | 0.649 | **61.749** | **1.161** |  |  | 3.509 | -0.350 | **4.627** | **0.161** |  |  |
| HG38 | 92.171 | 0.648 |  |  |  |  | 7.174 | -0.351 |  |  |  |  |
| HG39 | 72.723 | 0.649 | 69.336 | 0.912 | 57.824 | 0.837 | 9.048 | -0.350 | 10.290 | -0.088 | 9.422 | -0.163 |
| HG40 | 65.517 | 0.648 | 65.416 | 0.701 | 72.528 | 0.711 | 15.054 | -0.351 | 11.469 | -0.299 | 8.438 | -0.289 |
| HG41 | 62.677 | 0.648 | **62.065** | **0.963** | 75.636 | 0.350 | 18.896 | -0.351 | **8.444** | **-0.037** | 17.174 | -0.650 |
| HG42 |  |  | 79.927 | 0.519 |  |  |  |  | 11.282 | -0.481 |  |  |
| HG43 |  |  | **72.558** | **0.658** |  |  |  |  | **15.756** | **-0.342** |  |  |
| HG44 |  |  | 68.652 | 0.844 | 81.695 | 0.822 |  |  | 8.663 | -0.156 | 4.069 | -0.178 |
| HG45 |  |  | 64.683 | 0.816 | 78.494 | 0.581 |  |  | 14.147 | -0.184 | 12.404 | -0.419 |
| HG46 |  |  | **100.783** | **0.954** |  |  |  |  | **6.413** | **-0.046** |  |  |
| HG47 |  |  | 67.156 | 0.667 | 99.070 | 0.595 |  |  | 21.853 | -0.333 | 6.899 | -0.405 |
| HG48 |  |  | 79.196 | 0.832 |  |  |  |  | 9.546 | -0.168 |  |  |
| HG49 |  |  | 79.633 | 0.914 |  |  |  |  | 7.267 | -0.086 |  |  |
| HG50 |  |  | 84.589 | 0.856 |  |  |  |  | 6.751 | -0.144 |  |  |
| HG51 | 108.491 | 0.649 |  |  |  |  | 0.168 | -0.351 |  |  |  |  |

**Supplemental References**

Andrews, R. D., Jones, D. R., Williams, J. D., Thorson, P. H., Oliver, G. W., Costa, D. P., & Le Boeuf, B. J. (1997). Heart rates of Northern elephant seals diving at sea and resting on the beach. Journal of Experimental Biology, 200(15), 2083–2095. Retrieved from http://jeb.biologists.org/content/200/15/2083.abstract%5Cnpapers2://publication/uuid/0AC84255-C6A9-4252-B49C-A58508C079CF

Bennett, K. A., Robinson, K. J., Moss, S. E. W., Millward, S., & Hall, A. J. (2017). Using blubber explants to investigate adipose function in grey seals: glycolytic, lipolytic and gene expression responses to glucose and hydrocortisone. Scientific Reports, 7(1), 1–11. doi: 10.1038/s41598-017-06037-x

Boyd, I. L., Bevan, R. M., Woakes, A. J., & Butler, P. J. (1999). Heart rate and behavior of fur seals: Implications for measurement of field energetics. American Journal of Physiology - Heart and Circulatory Physiology, 276(3 45-3), 844–857. doi: 10.1152/ajpheart.1999.276.3.h844

Boyd, I. L., Woakes, A. J., Butler, P. J., Davis, R. W., & Williams, T. M. (1995). Validation of heart rate and doubly labelled water as measures of metabolic rate during swimming in California sea lions. Functional Ecology, 9, 151–160.

Brannan, N. B. L. (2017). *Investigating the physiological underpinnings of proactive and reactive behavioural types in grey seals (*Halichoerus grypus*)*. Durham University.

Halsey, L. G., Green, J. A., Twiss, S. D., Arnold, W., Burthe, S. J., Butler, P. J., … Careau, V. (2019). Flexibility, variability and constraint in energy management patterns across vertebrate taxa revealed by long‐term heart rate measurements. Functional Ecology, 33(2), 260–272. doi: 10.1111/1365-2435.13264

Lester, C. W., & Costa, D. P. (2006). Water conservation in fasting northern elephant seals (Mirounga angustirostris). Journal of Experimental Biology, 209(21), 4283–4294. doi: 10.1242/jeb.02503

Mellish, J.-A. E., Iverson, S. J., & Bowen, W. D. (2000). Metabolic compensation during high energy output in fasting, lactating grey seals (*Halichoerus grypus*): metabolic ceilings revisited. Proceedings of the Royal Society B-Biological Sciences, 267(1449), 1245–1251. doi: 10.1098/rspb.2000.1134

Pomeroy, P. P., Anderson, S. S., Twiss, S. D., & McConnell, B. J. (1994). Dispersion and site fidelity of breeding female grey seals (Halichoerus grypus) on North Rona, Scotland. Journal of Zoology, 233(3), 429–447. doi: 10.1111/j.1469-7998.1994.tb05275.x

Pomeroy, P. P., Twiss, S. D., & Duck, C. D. (2000). Expansion of a grey seal (*Halichoerus grypus*) breeding colony: changes in pupping site use at the Isle of May, Scotland. Journal of Zoology, 250, 1–12. doi: 10.1111/j.1469-7998.2000.tb00573.x

Ponganis, P. J., Stockard, T. K., Levenson, D. H., Berg, L., & Baranov, E. A. (2006). Cardiac output and muscle blood flow during rest-associated apneas of elephant seals. Comparative Biochemistry and Physiology - A Molecular and Integrative Physiology, 144(1), 105–111. doi: 10.1016/j.cbpa.2006.02.009

Robinson, K. J., Hall, A. J., Debier, C., Eppe, G., Thomé, J. P., & Bennett, K. A. (2018). Persistent Organic Pollutant Burden, Experimental POP Exposure, and Tissue Properties Affect Metabolic Profiles of Blubber from Gray Seal Pups. Environmental Science and Technology, 52(22), 13523–13534. doi: 10.1021/acs.est.8b04240

Robinson, K. J., Hall, A. J., Scholl, G., Debier, C., Thomé, J. P., Eppe, G., … Bennett, K. A. (2019). Investigating decadal changes in persistent organic pollutants in Scottish grey seal pups. Aquatic Conservation: Marine and Freshwater Ecosystems, 29(S1), 86–100. doi: 10.1002/aqc.3137

Sami, S., Mikko, S., & Antti, K. (2004). Artefact correction for heart beat interval data. *Advanced Methods for Processing Bioelectrical Signals*, 1–10. Retrieved from https://www.firstbeat.com/app/uploads/2015/10/saalasti_et_al_probisi_2004_congress.pdf

Shuert, C. R., Pomeroy, P. P., & Twiss, S. D. (2018). Assessing the utility and limitations of accelerometers and machine learning approaches in classifying behaviour during lactation in a phocid seal. Animal Biotelemetry, 6(1), 14. doi: 10.1186/s40317-018-0158-y

Shuert, C. R., Pomeroy, P. P., & Twiss, S. D. (2020). Coping styles in capital breeders modulate behavioural trade-offs in time allocation : assessing fine-scale activity budgets in lactating grey seals (Halichoerus grypus) using accelerometry and heart rate variability. Behavioral Ecology and Sociobiology, 74(8). doi: 10.1007/s00265-019-2783-8

Smout, S., King, R. & Pomeroy, P. (2011) Integrating heterogeneity of detection and mark loss to estimate survival and transience in UK grey seal colonies. Journal of Applied Ecology, 48, 364–372. [doi: 10.1111/j.1365-2664.2010.01913.x](https://doi.org/10.1111/j.1365-2664.2010.01913.x)

Twiss, S. D., Caudron, A., Pomeroy, P. P., Thomas, C. J., & Mills, J. P. (2000). Finescale topographical correlates of behavioural investment in offspring by female grey seals, *Halichoerus grypus*. Animal Behaviour, 59(2), 327–338. doi: 10.1006/anbe.1999.1320

Twiss, S. D., Shuert, C. R., Brannan, N., Bishop, A. M., & Pomeroy, P. P. (2020). Reactive stress-coping styles show more variable reproductive expenditure and fitness outcomes. Scientific Reports, 10, 9550. doi: 10.1038/s41598-020-66597-3
